# Retrospective study on the impact of ulnar nerve dislocation on the pathophysiology of ulnar neuropathy at the elbow

**DOI:** 10.7717/peerj.6972

**Published:** 2019-05-20

**Authors:** Seok Kang, Joon Shik Yoon, Seung Nam Yang, Hyuk Sung Choi

**Affiliations:** Department of Rehabilitation Medicine, Korea University Guro Hospital, Seoul, South Korea

**Keywords:** Ulnar neuropathy at the elbow, Ulnar nerve displacement, Subluxation, Dislocation, Axonal loss, Demyelination

## Abstract

**Introduction:**

High resolution ultrasonography (US) has been used for diagnosis and evaluation of entrapment peripheral neuropathy. Ulnar neuropathy at the elbow (UNE) is the second most common focal entrapment neuropathy. The ulnar nerve tends to move to the anteromedial side and sometimes subluxates or dislocates over the medial epicondyle as the elbow is flexed. Dislocation of the ulnar nerve during elbow flexion may contribute to friction injury. We aimed to investigate the effects which the dislocation of ulnar nerve at the elbow could have on the electrophysiologic pathology of UNE.

**Materials:**

We retrospectively reviewed 71 arms of UNE. The demographic data, electrodiagnosis findings and US findings of ulnar nerve were analyzed. We classified the electrodiagnosis findings of UNE into three pathologic types; demyelinating, sensory axonal loss, and mixed sensorimotor axonal loss. The arms were grouped into non-dislocation, partial dislocation, and complete dislocation groups according to the findings of nerve dislocation in US examination. We compared the electrodiagnosis findings, ulnar nerve cross sectional areas in US and electrodiagnosis pathology types among the groups.

**Results:**

A total of 18 (25.3%) arms showed partial dislocation, and 15 (21.1%) arms showed complete dislocation of ulnar nerve in US. In the comparison of electrodiagnosis findings, the partial and complete dislocation groups showed significantly slower conduction velocities and lower amplitudes than non-dislocation group in motor conduction study. In the sensory conduction study, the conduction velocity was significantly slower in partial dislocation group and the amplitude was significantly lower in complete dislocation group than non-dislocation group. In the comparison of US findings, patients in partial and complete dislocation groups showed significantly larger cross sectional areas of the ulnar nerve. The comparison of electrodiagnosis pathologic types among the groups revealed that there were significantly larger proportions of the axonal loss (sensory axonal loss or mixed sensorimotor axonal loss) in partial and complete dislocation groups than non-dislocation group.

**Conclusion:**

The ulnar nerve dislocation could influence on the more severe damage of the ulnar nerve in patients with UNE. It might be important to evaluate the dislocation of the ulnar nerve using US in diagnosing ulnar neuropathy for predicting the prognosis and determining the treatment direction of UNE.

## Introduction

High resolution ultrasonography (US) has been used for diagnosis and evaluation of entrapment peripheral neuropathy (Klauser et al., 2009; Wiesler et al., 2006a, 2006b). Although the electrodiagnosis is the standard test in diagnosis of entrapment neuropathy, the US could provide the investigators additional informations. When investigating peripheral neuropathy, the US could assess the size, shape, and echo-texture of the affected nerves (Jacobson, Wilson & Yang, 2016; Suk, Walker & Cartwright, 2013). The US also has the advantage of performing dynamic scans as well as static scans (Jacobson et al., 2001; Martinoli et al., 2001; Ozturk et al., 2008).

Ulnar neuropathy at the elbow (UNE) is the second most common focal entrapment neuropathy after carpal tunnel syndrome (Osei et al., 2017; Staples & Calfee, 2017). During elbow flexion, the cubital tunnel ligament is tightened and has the potential to compress the ulnar nerve beneath the humeroulnar aponeurosis. In addition, the ulnar nerve tends to move to the anteromedial side and sometimes subluxates or dislocates over the medial epicondyle as the elbow is flexed (Okamoto et al., 2000; Yang et al., 2013). Dislocation of the ulnar nerve during elbow flexion may contribute to friction injury (Tsujino & Ochiai, 2001) and be a predisposing factor for UNE. It has been reported that the ulnar nerve dislocation was associated with tardy ulnar palsy in cubitus varus deformity (Jeon et al., 2006). A previous case study had suggested that the abnormal dislocation of the ulnar nerve during elbow flexion increased the possibility of its mechanical injury (Lewanska, Grzegorzewski & Walusiak-Skorupa, 2016). The dynamic US scan is an optimal tool for the assessment of ulnar nerve dislocation (Chuang et al., 2016).

The ulnar nerve dislocation could be observed even in healthy individuals (Calfee et al., 2010; Childress, 1956; Okamoto et al., 2000; Ozturk et al., 2008). In previous study, the patients with UNE showed significantly greater degree of ulnar nerve movement (Yang et al., 2013). However, there have been few studies that investigated the relation between the ulnar nerve dislocation and electrophysiologic pathology of UNE. In this study, we aimed to investigate the effects which the dislocation of ulnar nerve at the elbow could have on the electrophysiologic pathology of UNE.

## Method

This was a retrospective observational study conducted on the consecutive patients who underwent electrophysiologic study and US for diagnosis of UNE between January 2013 and December 2015. The inclusion criteria were as follows: (1) typical clinical signs and symptoms indicating ulnar nerve lesion (i.e., paresthesia and/or hypesthesia on the fourth and fifth digits of hand, weakness and atrophy of ulnar innervated hand muscles, tinel signs at the elbow), (2) definite electrodiagnosis findings of UNE. We excluded the patients who met the following criteria: (1) traumatic ulnar nerve lesion, (2) ulnar nerve entrapment in other sites such as wrist, (3) perviously operated UNE, (4) polyneuropathy, (5) C8-T1 cervical radiculopathy, (6) lower trunk brachial plexopathy. We had enrolled twenty arms of 11 subjects who had never experienced symptoms or clinical signs of ulnar neuropathy, as control group. This study was approved by the Institutional Review Board of the Korea University Guro Hospital (2017GR0806). The informed consent from participants was waived by Institutional Review Board.

### Electrodiagnosis

The electrodiagnosis of UNE included the motor and sensory nerve conduction studies (NCS) of ulnar and dorsal ulnar cutaneous nerves and the needle electromyography of abductor digiti minimi (ADM), first dorsal interossei, and flexor carpi ulnaris muscles. The compound motor action potentials (CMAP) were recorded from the ADM muscle. The ulnar nerve short-segmental study was performed in across-the-elbow segment (between three cm below and seven cm above the medial epicondyle). The elbow was extended in wrist stimulation, and flexed 90° in across-the-elbow segmental study. The recording sites of antidromic sensory conduction studies were the fifth finger and the fourth web space of hand in the ulnar and the dorsal ulnar cutaneous nerve stimulations, respectively.

Diagnosis of UNE was made according to diagnostic criteria proposed by the American Association of Electrodiagnostic Medicine (AAEM) (Campbell et al. 1999).

- Absolute motor nerve conduction velocity from above elbow (AE) to below elbow (BE) of less than 50 m/s.
- An AE-to-BE segment greater than 10 m/s slower than the BE-to-wrist segment.
- A decrease in CMAP negative peak amplitude from BE to AE greater than 20%.
- A significant change in CMAP configuration at the AE site compared to the BE site.

We classified the electrodiagnosis findings of UNE into three pathologic types (Scheidl et al., 2013). The demyelinating type UNE was defined as that the CMAP across the elbow revealed significant slowing of conduction velocity (≥10 m/s in comparison to the forearm), with or without motor conduction block (CMAP amplitude reduction ≥20%), and both sensory nerve action potential (SNAP) and CMAP with distal stimulation (wrist) showed the amplitude within normal limits. The sensory axonal type UNE was defined as that the SNAP was of low amplitude (<10 μV). The mixed sensorimotor axonal type was defined as both SNAP and CMAP were of low amplitudes (<10 μV and <4 mV, respectively) with distal stimulation, and abnormal spontaneous activities (positive sharp waves or fibrillation potentials) were observed in ulnar innervated muscles.

### Ultrasonography

For ultrasound examinations, a HD15 ultrasound device (Philips Healthcare, Bothell, WA, USA) with a 5–12 MHz linear array transducer was used. The examinations were performed by a physician who had more than 10 years of experiences in musculoskeletal US. During the examination, the patients were placed in supine position. The transducer was placed at the medial epicondyle level and cross‐sectional images of the ulnar nerve and the cubital tunnel were taken. In transverse plane, the nerve was traced from the inlet to the outlet of cubital tunnel. The nerve cross-sectional areas (CSAs) were measured at the maximum swelling point. The examiner carefully placed the probe perpendicular to the nerve to obtain the most accurate CSA. The CSA was measured using automatic manual “tracing” just inside the hyperechogenic line that surrounds the nerve perineurium. In each US examination, the examiner measured CSA three times and the mean value was recorded to minimize error.

**Figure 1:**
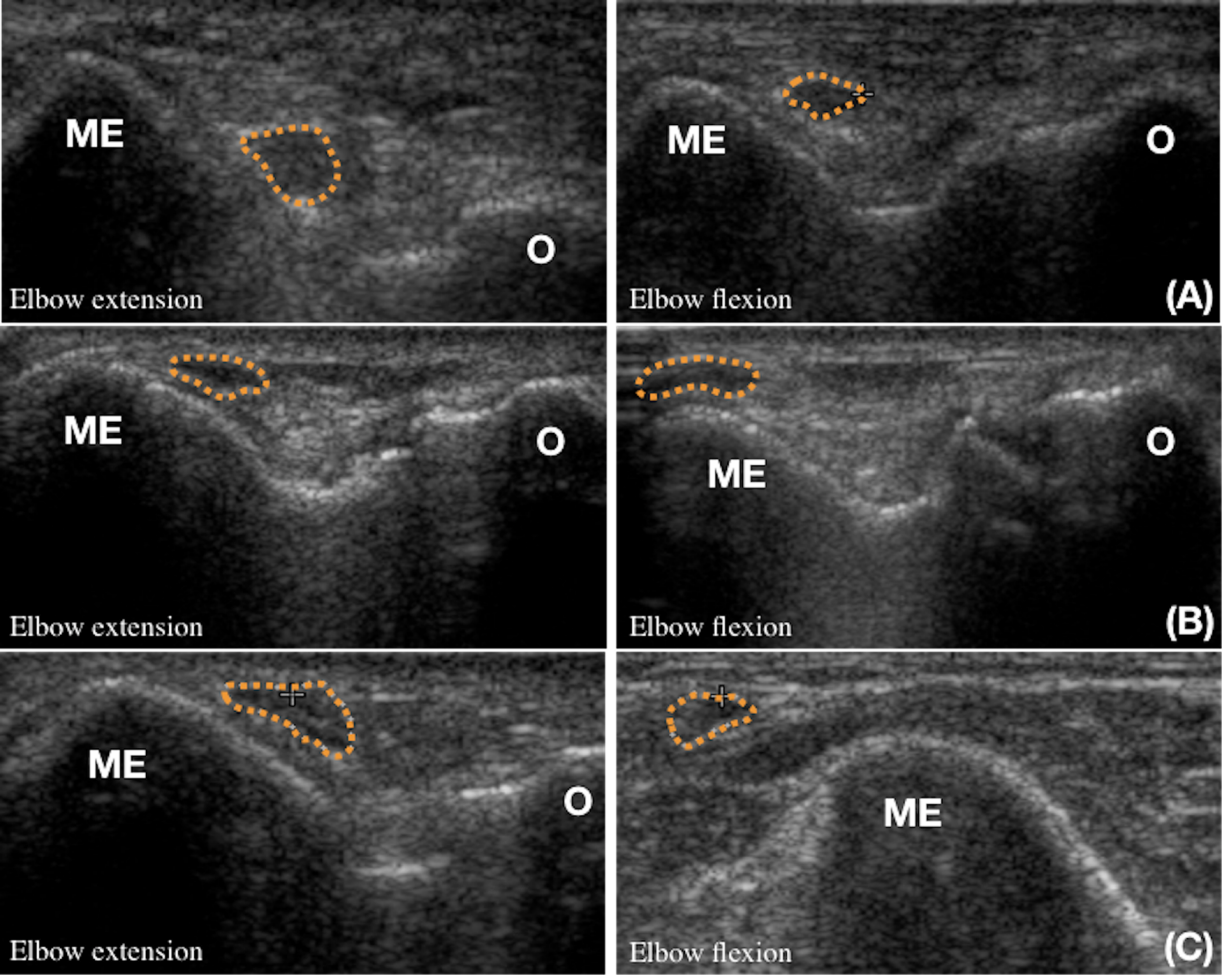
The ultrasonographic classifications of ulnar nerve (dotted line) displacement during elbow flexion. (A) Non-dislocation of ulnar nerve. (B) Partial dislocation of ulnar nerve. (C) Complete dislocation of ulnar nerve. ME, medial epicondyle; O, olecranon.

For the evaluation of ulnar nerve dislocation, the elbow was imaged dynamically from full extension to full flexion. An effort was made to minimize transducer pressure on the nerve to minimize its effect on nerve movement. We classified the findings of ulnar nerve dislocation into three categories; non-dislocation, partial dislocation and complete dislocation (Fig. 1). The partial dislocation is defined as ulnar nerve moved on the tip of medial epicondyle during elbow flexion. The complete dislocation is defined as the nerve moved anteriorly beyond the tip of medial epicondyle. We grouped the patients according to the patterns of dislocation of ulnar nerve at elbow flexion.

### Statistical analysis

The analysis of variance was performed to compare the quantitative variables (body mass index (BMI), disease duration and age) of demographic data and the results of electrodiagnosis and ulnar nerve CSAs among the groups according to the ulnar nerve dislocation. Tucky’s test was used for the post hoc analysis. In the analysis of nominal variables (gender and site) of demographic data and the pathology types of neuropathy among the groups, we performed the chi-square analysis. Bonferroni correction was conducted for the intergroup multiple comparisons. In the comparisons between the control group and patients with UNE, we used the independent *t*-test for the quantitative variables (BMI, age, and CSAs), and the chi-square analysis for the nominal variables (gender, site, and ulnar nerve dislocation). We conducted the logistic regression analysis to investigate which factors affected the development of axonal loss of UNE. All *p*-values were two-sided, and a *p*-value of < 0.05 was considered to reflect statistical significance. After Bonferroni correction, *p* < 0.017 (0.05/3) was considered to denote statistical significance. The statistical analyses were conducted using the Statistical Package for the Social Sciences version 22.0 (IBM Corp., Armonk, NY, USA) software package for Windows.

## Result

We enrolled 71 arms (65 patients) of UNE in this study. A total of 38 (53.5%) arms were classified into non-dislocation group, 18 (25.4%) arms were partial dislocation group, and 15 (21.1%) arms were complete dislocation group. The demographic data of the arms are revealed in Table 1. There were no significant differences in the age, sex, and BMI among the groups. The disease duration and site of neuropathy were also not significantly different.

**Table 1:** Comparisons of demographic characteristics among the groups according to ulnar nerve dislocation in UNE patients.

Group	Non-dislocation (*N* = 38)	Partial dislocation (*N* = 18)	Complete dislocation (*N* = 15)	*p*-value
Age (years)	45.37 ± 13.73	48.33 ± 14.69	48.47 ± 13.55	0.661^a^
Male (%)	24 (63.2)	12 (66.7)	11 (73.3)	0.779^b^
Right side (%)	24 (63.2)	13 (72.2)	7 (46.7)	0.314^b^
Body mass index	22.68 ± 2.46	23.82 ± 3.71	22.02 ± 2.58	0.182^a^
Disease duration (months)	9.29 ± 11.47	11.24 ± 10.19	11.37 ± 9.92	0.739^a^

**Notes:**

a The *p*-values were calculated by ANOVA.

b The *p*-values were calculated by chi-square analysis.

Table 2 shows the comparisons of demographic data and ulnar nerve dislocation between the control group and patients with UNE. In control group, the partial dislocation was observed in 5 (25.0%) out of 20 arms, and the complete dislocation in 2 (10.0%) arms. There were no significant differences in the incidence of ulnar nerve dislocation between the control group and patients with UNE. However, the CSAs of ulnar nerve were significantly larger in patients with UNE.

**Table 2:** Comparisons of demographic characteristics and ultrasound findings of ulnar nerve between the control group and patients with UNE.

	Control (*N* = 20)	UNE patients (*N* = 71)	*p*-value
Age (years)	43.95 ± 16.00	46.77 ± 13.82	0.438^a^
Male (%)	12 (60.0%)	47 (66.2%)	0.608^b^
Right side (%)	11 (55.0%)	44 (62.0%)	0.573^b^
Body mass index	22.85 ± 1.67	22.83 ± 2.88	0.975^a^
Ulnar nerve dislocation			0.496^b^
None (%)	13 (65.0)	38 (53.5)	
Partial (%)	5 (25.0)	18 (25.4)	
Complete (%)	2 (10.0)	15 (21.1)	
Cross sectional area (mm^2^)	6.44 ± 1.02	14.72 ± 5.10	0.000^a^*

**Notes:**

* *p* < 0.05.

a The *p*-values were calculated by independent *t*-test.

b The *p*-values were calculated by chi-square analysis.

**Figure 2:**
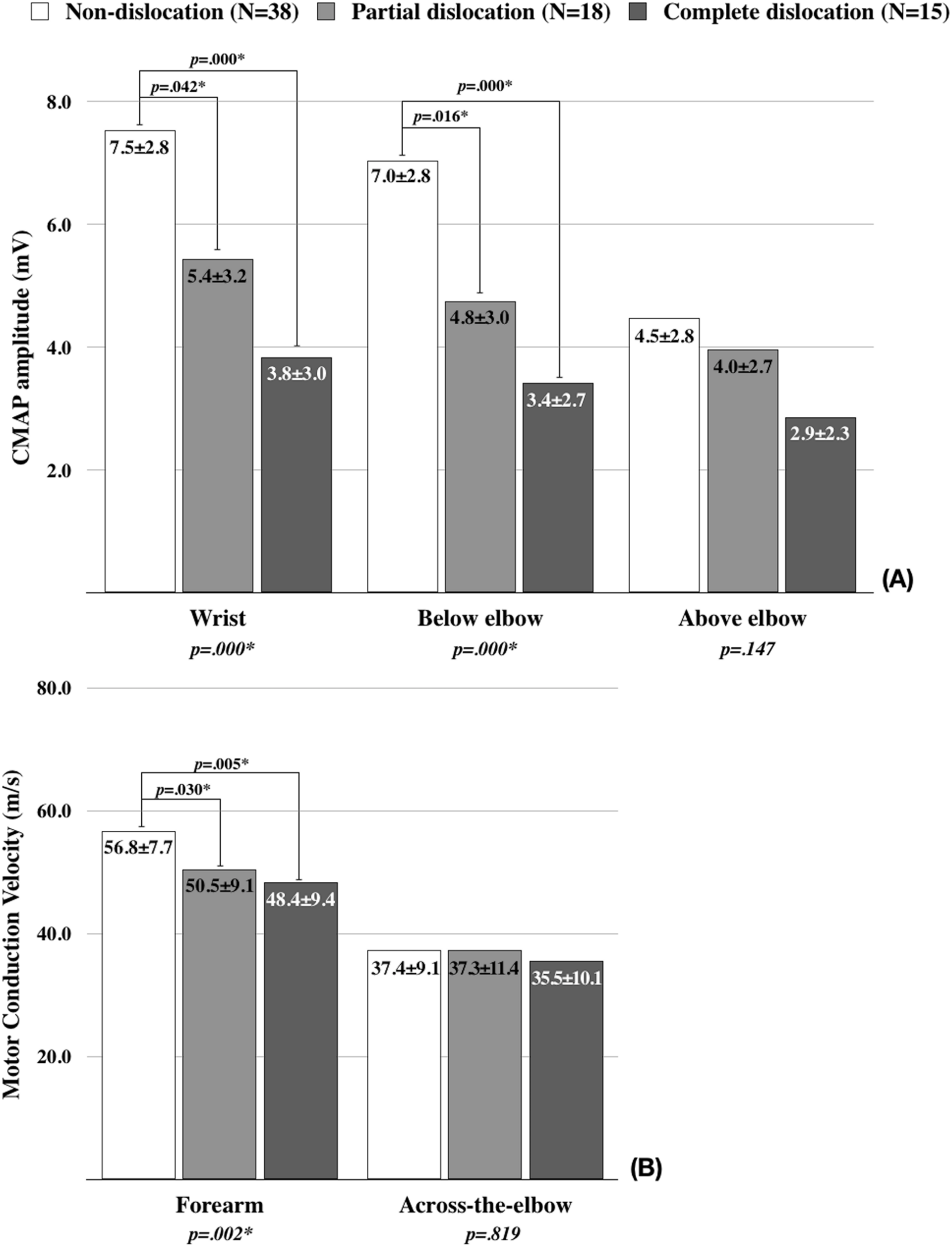
The comparison of motor nerve conduction study findings among the groups. (A) Comparison of compound muscle action potential (CMAP) amplitude. (B) Comparison of conduction velocity. *p*-values were calculated using ANOVA. Tucky’s test was used for the post hoc analysis. *p < 0.05.

The comparisons of motor NCS results among the groups are revealed in Fig. 2. There were significant differences in the CMAP amplitudes from stimulations at the wrist and below the elbow. In the post hoc analysis, the CMAP amplitudes were significantly lower in the partial and complete dislocation groups than the non-dislocation group (Fig. 2A). The motor conduction velocities of forearm segment were also significantly different among the groups. The post hoc analysis revealed that the conduction velocities were significantly lower in the partial and complete dislocation groups than the non-dislocation group (Fig. 2B).

**Figure 3:**
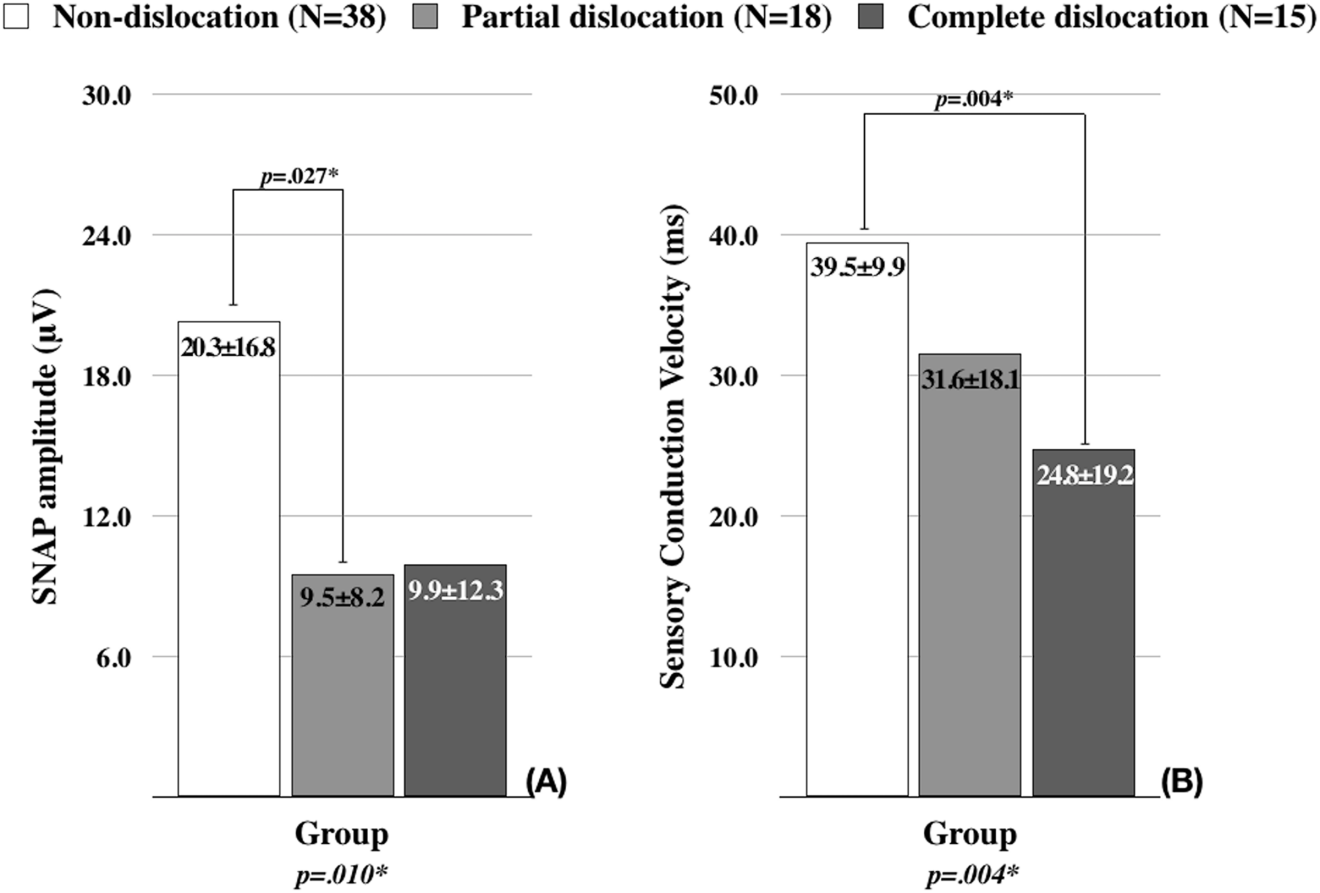
The comparison of sensory nerve conduction study findings among the groups. (A) Comparison of sensory nerve action potential (SNAP) amplitude. (B) Comparison of conduction velocity. *p*-values were calculated using ANOVA. Tucky’s test was used for the post hoc analysis. *p < 0.05.

The results of sensory NCS were significantly different among the groups, as presented in Fig. 3. The SNAP amplitudes and the sensory conduction velocities were lower in the patients with ulnar nerve dislocation (partial and complete dislocation groups) than non-dislocation group. The post hoc analysis revealed that the statistical significances were present between the partial dislocation and non-dislocation groups in the SNAP amplitudes (Fig. 3A), and between the complete dislocation and non-dislocation groups in the sensory conduction velocities (Fig. 3B).

**Figure 4:**
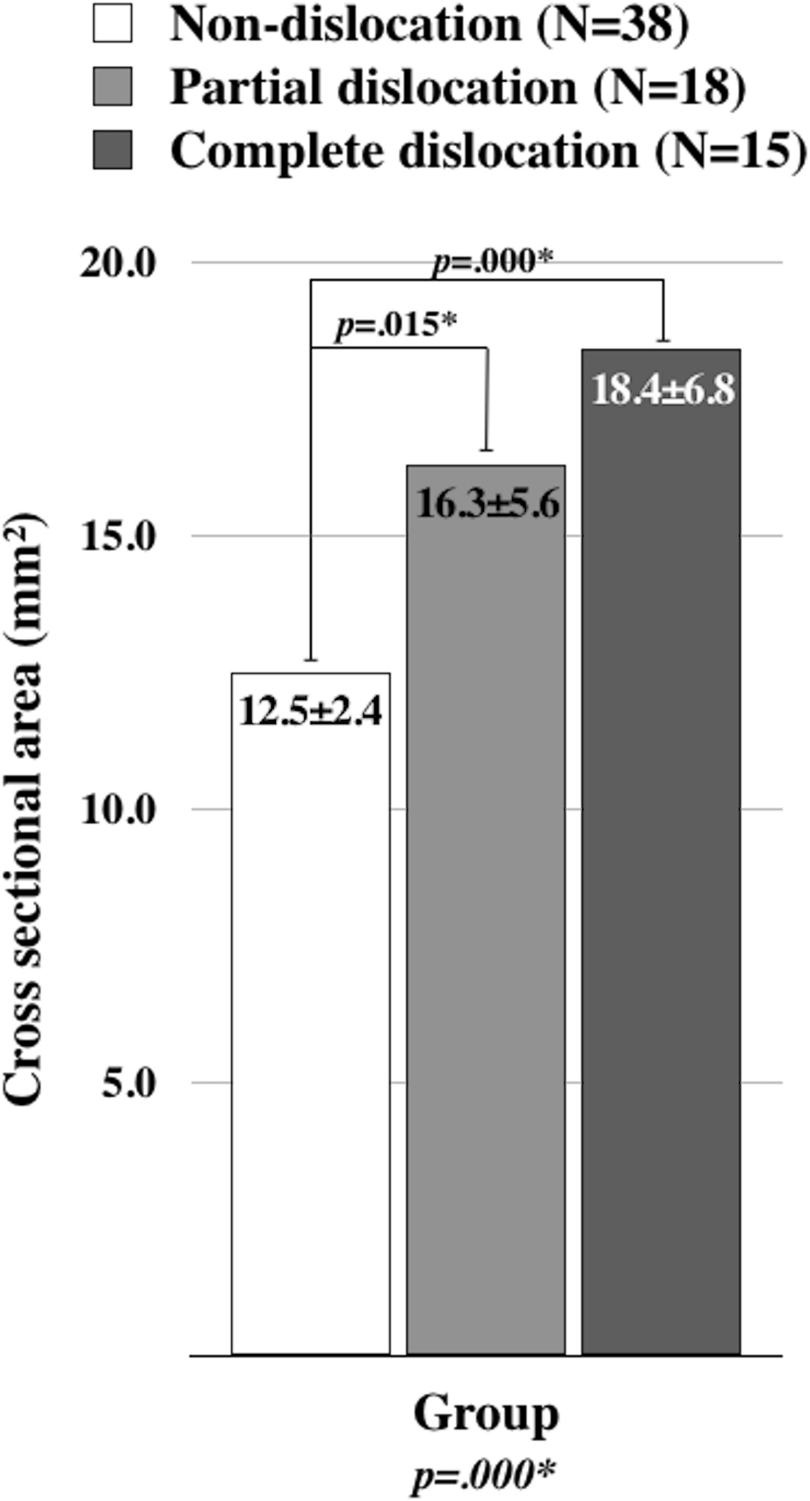
The comparison of ulnar nerve cross section area among the groups. *p*-values were calculated using ANOVA. Tucky’s test was used for the post hoc analysis. *p < 0.05.

**Figure 5:**
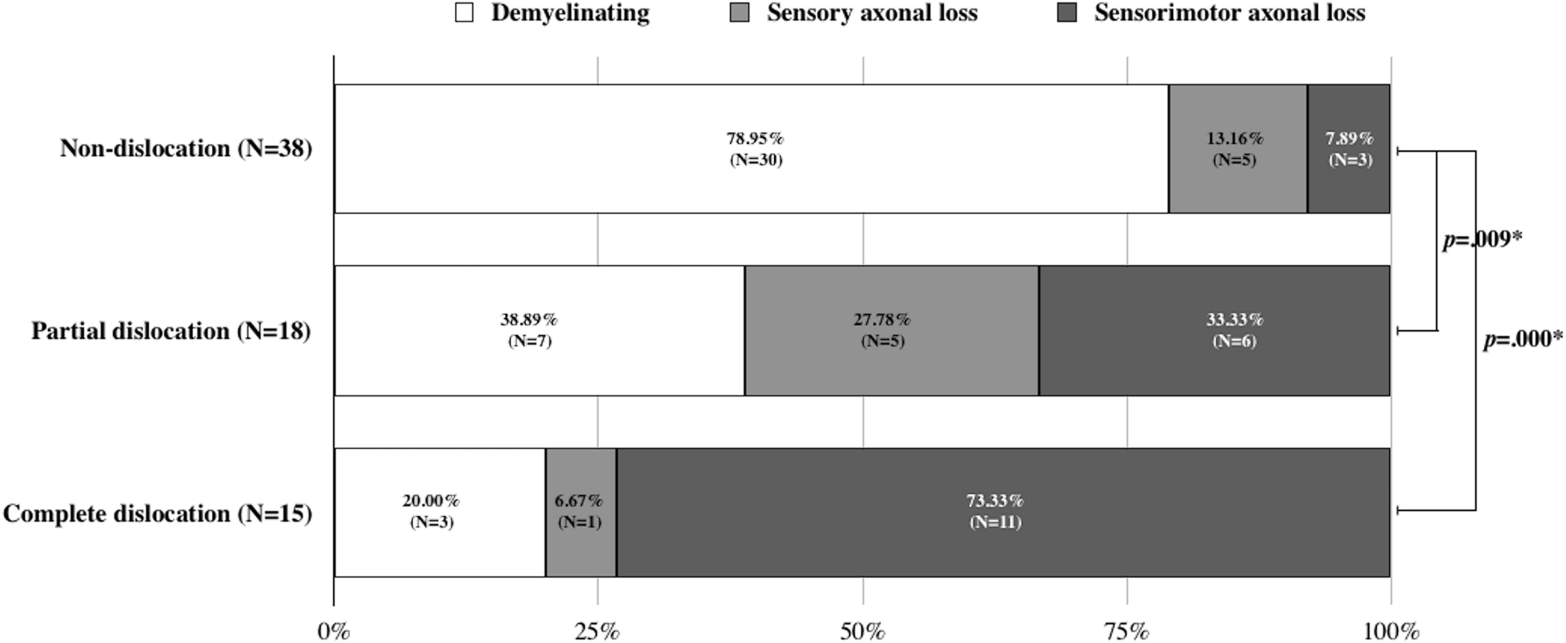
The relationship between the ulnar nerve displacement and the electrophysiologic pathology of ulnar neuropathy at the elbow. *p*-values were calculated using chi-square test. Bonferroni correction was conducted for the intergroup comparisons. *p < 0.017.

In Fig. 4, the CSAs of ulnar nerve were compared among the groups. The patients with ulnar nerve dislocation showed significantly larger CSAs of ulnar nerve than non-dislocation group. There was no significant difference between the partial and complete dislocation groups. The relationship between the ulnar nerve dislocation and the electrophysiologic pathology of UNE is presented in Fig. 5. The significant difference was observed in the proportion of pathologic types of UNE among the groups (*p* = 0.000). The intergroup comparisons after Bonferroni correction revealed that there were significantly larger numbers of sensory axonal loss and mixed sensorimotor axonal loss of UNE in the patients with ulnar nerve dislocation than non-dislocation group. However, there was no significant difference between the partial and complete dislocation groups.

The results of logistic regression analysis to investigate the factors contributing to the axonal type (sensory axonal or mixed sensorimotor axonal type) UNE are shown in Table 3. The dislocation of ulnar nerve was the significant independent factor contributing to the axonal loss of UNE (*p* = 0.000). All the other factors were not statistically significant.

**Table 3:** Logistic regression analysis of factors contributing to axonal loss of UNE.

Factor	Odd ratio	95% confidence interval		*p*-value
Dislocation	8.625	2.939	25.316	0.000*
Gender (male)	2.534	0.887	7.239	0.083
Side (right)	1.710	0.649	4.506	0.277
Age	1.036	0.998	1.074	0.060
Disease duration	1.010	0.967	1.055	0.655
Body mass index	0.971	0.823	1.145	0.726

**Note:**

* *p* < 0.05.

## Discussion

We have investigated the relation between the ulnar nerve dislocation and the pathophysiology of UNE. In this study, more axonal loss of ulnar nerve was observed in patients with dislocation or subluxation of the ulnar nerve at elbow flexion. Not only in the ultrasonographic findings but also the electrophysiologic findings, the ulnar nerve dislocation could influence on the more severe damage of the ulnar nerve in patients with UNE. These results suggest that the dislocation of ulnar nerve at the elbow is related with the pathophysiology of UNE.

The ulnar nerve dislocation had been investigated in many previous studies. The ulnar nerve dislocation had been observed in 16.2% out of two thousand elbows according to a study of Childress (1956). Calfee et al. (2010) had reported that the ulnar nerve hypermobility was identified in 37% of 400 elbows. Repetitive friction and shear stress due to hypermobility of ulnar nerve may be a cause of UNE (Jeon et al., 2006; Michelin et al., 2017; Pham & Gupta, 2009; Tsujino & Ochiai, 2001).

As the development of US enables the dynamic examination, several studies in which the nerve dislocation was investigated using US have been published. Okamoto et al. (2000) had examined 200 normal elbows using US. They reported that the partial dislocation of ulnar nerve was observed in 27% and the complete dislocation was observed in 20%. In another study, the incidence rates of ulnar nerve partial and complete dislocation in 212 elbows of healthy volunteers were 23.1% and 8.5%, respectively (Ozturk et al., 2008). In our study, the control group showed partial dislocation in 25% and complete dislocation in 10%, similar to those previous studies.

The difference in the incidence rates of ulnar nerve dislocation between the healthy subjects and patients with UNE has not been established yet (Lewanska, Grzegorzewski & Walusiak-Skorupa, 2016). Previous studies of investigating the ulnar nerve dislocation in patients with UNE reported that the ulnar nerve partial and complete dislocation were occurred in 14–18.7% and the 6.7–9.9%, respectively (Filippou et al., 2010; Van Den Berg et al., 2013). In the study of Van Den Berg et al. (2013), there were no significant differences in the presence of ulnar nerve partial and complete dislocation between healthy controls and patients with UNE. In addition, Omejec & Podnar (2016a) had reported that ulnar nerve dislocations tended to be more common in controls compared with UNE patients. They concluded that ulnar nerve dislocation does not cause symptomatic UNE. By contrast, in a recent study of investigating the 234 elbows (89 with UNE and 145 control), there were significantly higher rates of ulnar nerve dislocation in elbows with UNE compared to controls (partial dislocation 24% vs 12% and complete dislocation 24% vs 11%) (Schertz et al., 2017). In our study, similar incidence rates of ulnar nerve dislocation were observed in patients with UNE (partial dislocation in 25.4% and complete dislocation in 21.1%). However, there were no significant differences between the control group and patients with UNE.

There have been some studies that investigated the US findings according to the severity of UNE. In the study of Bayrak et al. (2010), there was a statistically significant correlation between the largest CSA of ulnar nerve and electrophysiologic severity of UNE and the largest CSA was the most valuable US measurement for diagnosis and determining the severity of UNE. Scheidl et al. (2013) had studied the relation between the US findings of ulnar nerve and the types of pathology of UNE. They reported that the ulnar nerve CSA was significantly larger in axonal type than demyelinating type (15.2 ± 5.8 vs 10.1 ± 2.6 mm^2^) and US findings could reflect the type and severity of UNE. In our study, the similar findings were observed in comparison of the ulnar nerve CSA between the axonal group (sensory axonal and mixed sensorimotor axonal groups, *n* = 31) and demyelinating group (*n* = 40). The analysis using an independent *t*-test showed that the CSA of axonal group was significantly larger than demyelinating group (17.2 ± 6.3 vs 12.8 ± 2.6 mm^2^, *p* = 0.000).

In present study, the pathologic type of UNE was significantly different according to the ulnar nerve dislocation. We had classified the severity of UNE as three levels; demyelinating, sensory axonal, and sensorimotor axonal. Difference between sensory and sensorimotor axonal loss is most probably due to absent collateral reinnervation on the sensory side. There were a significantly larger number of patients with more severe type of UNE in more severe ulnar nerve dislocation group. In the comparison of electrophysiologic findings according to the ulnar nerve dislocation, the patients in partial or complete dislocation group revealed significantly worse findings in multiple electrodiagnostic parameters than non-dislocation group. Not only CMAP and SNAP amplitudes, but also there were significant differences of sensory nerve conduction velocities between the complete dislocation and non-dislocation groups. Because the axonal losses occur more predominantly in large size fibers, the sensory conduction velocity decrements in complete dislocation group may be explained by the axonal losses of large and fast nerve fibers. The ulnar nerve CSAs were also significantly larger in ulnar nerve partial and complete dislocation groups than non-dislocation group. These findings may indicate that the ulnar nerve dislocation could effect on the severity or pathologic type of UNE. There are still many controversies against the association between the development of UNE and the incidence of ulnar nerve dislocation. However, at least, the results of this study suggest that the dislocation of ulnar nerve in patients with UNE could related with the aggravation of electrodiagnostic findings. It could be thought that the repetitive friction and shear stress additional to the compression due to hypermobility of ulnar nerve would induce deterioration of neuropathy.

On the contrary to our study findings, Van Den Berg et al. (2013) had reported that the electrodiagnostic and sonographic findings did not differ between the patients with and without ulnar nerve dislocation. This difference is probably due to the electrodiagnostic criteria of UNE. In our study, we reviewed the electrodiagnostic data of patients with UNE strictly confirmed in accordance with the diagnostic criteria proposed by AAEM. In the study of Van Den Berg et al. (2013) the mean motor conduction velocity in across-the-elbow segment was 50.0 m/s in patients with or without ulnar nerve dislocation. The mean reductions of CMAP amplitude were only 4.2% and 3.1% in non-dislocation and dislocation groups, respectively. However, the AAEM criteria suggest the absolute motor nerve conduction velocity in across-the-elbow segment of less than 50 m/s, the slowing of more than 10 m/s than forearm segment, and a decrease in CMAP amplitude from BE to AE greater than 20%. All the patients of our study, in regardless of ulnar nerve dislocation, showed that the mean motor conduction velocity in across-the-elbow segment and decrease of CMAP amplitude were 36.9 m/s and 25.0%, respectively. Because the aim of this study is to investigate the impact of dislocation of ulnar nerve on the electrophysiologic findings, the absolute application of definite criteria for the pathology in electrodiagnosis may be critical.

Recently, two studies had been published arguing against the role of ulnar nerve dislocation in UNE. Leis et al., had reported that UNE occurs less frequently and is less severe on the side of complete ulnar nerve dislocation. They concluded that complete dislocation may even have a protective effect on the ulnar nerve (Leis et al., 2017). The other study (Omejec & Podnar, 2016a), and additional reply (Podnar & Omejec, 2016) suggested that partial ulnar nerve dislocation might cause mild UNE, and that entrapment under the humeroulnar aponeurosis predisposes the ulnar nerve to complete dislocation. However, in our study, the findings of UNE in ulnar nerve dislocation group revealed more severe UNE. We think that the more severe findings of UNE with ulnar nerve dislocation group might be the result of additional friction injury of ulnar nerve during elbow flexion in combination with the entrapment under the humeroulnar aponeurosis.

In previous studies, UNE under the humeroulnar aponeurosis were mainly axonal and more severe (Omejec & Podnar, 2015, 2016b), and with higher incidence of complete dislocation (Omejec & Podnar, 2016a). It might be hypothesized that the entrapment prevents the ulnar nerve gliding during elbow flexion and could cause traction force on the nerve to be dislocated over the medial epicondyle. At least the results of our study suggest that partial or complete dislocation of ulnar nerve during elbow flexion are related with aggravating factor of UNE. We think that ulnar neuropathy could be aggravated by friction force caused by dislocation over medial epicondyle during repetitive elbow flexion and extension. The nerve injury could also be deteriorated by repetitive external compressive force to the dislocated ulnar nerve occurred in a posture such as putting weight on the flexed elbow. In partial dislocation, the ulnar nerve might be more susceptible to external compressions. We think that the ulnar nerves of patients with UNE might be vulnerable to the additional friction and compression injuries. Thus, if ulnar entrapment neuropathy would have occurred, the ulnar nerve might be vulnerable to the additional mechanism of injuries than normal nerves and the dislocation of ulnar nerve could act as a deteriorating factor. However, in this study it might be uncertain whether the ulnar nerve dislocation caused severe type of ulnar neuropathy or the dislocation was caused by severe entrapment. Further prospective studies should be needed.

There are several limitations in this study. First, this study was conducted in a retrospective design. We have reviewed the data of consecutive patients by a single examiner, nonetheless there is a possibility of selection bias. In addition, because of the retrospective design, we could not examine more precise localization of ulnar neuropathy. Second, while the US examination was performed by a single examiner, the electrodiagnostic tests were performed by multiple examiners. In spite that the electrodiagnosis has relatively low risk of examiner dependency, the reliability could be lower than the study by a single examiner. Third, the severity of specific clinical symptoms cannot be analyzed due to limitation of recorded information. If further prospective studies could be planned, the clinical data of specific symptoms, such as parestheisa and weakness, should be included in a study design and analysis. Lastly, the patients included in study were small in number. We have included 71 patients. Compared to previous studies, these numbers are relatively small. Further large-sized studies are needed.

## Conclusions

Despite the retrospective study, demographic data such as age, disease duration, sex, and BMI of the patients included in this study did not differ between groups according to ulnar nerve dislocation. Therefore, the findings of this study are meaningful in understanding the impact of ulnar nerve dislocation on electrophysiologic pathology of ulnar neuropathy. In particular, the results of logistic regression analysis indicate that the dislocation of ulnar nerve could be an important factor contributing to axonal loss in UNE. The ulnar nerve dislocation could influence on the more severe damage of the ulnar nerve in patients with UNE. Considering these findings, it might be important to evaluate the dislocation of the ulnar nerve using US in diagnosing ulnar neuropathy for predicting the prognosis and determining the treatment direction of UNE.

## Supplemental Information

10.7717/peerj.6972/supp-1 Supplemental Information 1 Demographic data, nerve conduction study data and ultrasonography data.

## Additional Information and Declarations

### Data Availability

The following information was supplied regarding data availability: The raw measurements are available in the Supplemental File.
